# Supplementary material for: Tolerogenic Dendritic Cells Induce Apoptosis-Independent T Cell Hyporesponsiveness of SARS-CoV-2-Specific T Cells in an Antigen-Specific Manner
Source: Int J Mol Sci. 2022 Dec 2;23(23):15201. doi: 10.3390/ijms232315201 (PMC9740551; doi:10.3390/ijms232315201)
Supplement: Supplementary file 1 [file ijms-23-15201-s001.zip › ijms-2055337-supplementary.pdf]

SUPPLEMENTARY MATERIAL

Supplementary table S1: Information on antibodies used in the current study. Specificity, fluorochrome, clone, reference number and company are provided for each antibody or dye used, when applicable.

Marker	Fluorochrome	Clone	Reference number	Company
tolDC and convDC phenotype analysis				
CD209	FITC	DCN46	551264	BD Pharmingen™
HLA-DR	PE	L243	347401	BD™
CD14	PerCP	MϕP9	345786	BD™
CD80	PECy5	L307.4	559370	BD Pharmingen™
CD83	FITC	HB15e	MHCD8301	Life Technologies™
CD86	PE	IT2.2	555665	BD Pharmingen™
IgG isotype	FITC	X40	345815	BD™
IgG isotype	PE	X40	345816	BD™
IgG isotype	PerCP	X40	345817	BD™
SARS-CoV-2-specific T cell sort				
CD3	PerCPCy5.5	UCHT1	300430	Biolegend
CD4	BV510	RPA-T4	300546	Biolegend
CD8	PB	SK1	344718	Biolegend
CD71	BV786	M-A712	563768	Biolegend
CD98	BB515	UM7F8	565103	Biolegend
LIVE/DEAD Fixable Near-IR Dead Cell Stain Kit	NA	NA	L10119	Thermo Fisher Scientific
DC – T cell coculture follow-up				
CD3	PECy7	UCHT1	563423	Biolegend
CD4	APC-H7	RPA-T4	560158	Biolegend
CD8	PB	SK1	344718	ThermoFisher™
Annexin-V	APC	NA	640941	Biolegend
CellEvent™ Caspase-3/7 Green Flow Cytometry Assay Kit	FITC	NA	C10427	Invitrogen™
Viability (Cytoflex)				
PI	NA	NA	P3566	Thermo Fisher Scientific

Supplementary table S2: Expression levels of cellular markers HLA-DR, CD80, CD83 and CD86 show a significant decrease in tolDC as shown by MFI values.

Marker	Celltype	MFI ± SD	Decrease (%) ± SD (%)
HLA-DR	convDC	$2.21*10^{6} \pm 7.08*10^{5}$	73.47 ± 9.70
	tolDC	$5.56*10^{5} \pm 2.21*10^{5}$	
CD80	convDC	$9.72*10^{4} \pm 3.20*10^{4}$	57.79 ± 22.27
	tolDC	$3.33*10^{4} \pm 1.56*10^{4}$	
CD83	convDC	$1.75*10^{5} \pm 2.71*10^{4}$	69.54 ± 10.42
	tolDC	$4.75*10^{4} \pm 1.48*104$	
CD86	convDC	$3.40*10^{6} \pm 5.35*10^{5}$	65.68 ± 10.87
	tolDC	$1.07*10^{6} \pm 2.71*10^{5}$	

Supplementary table S3: longitudinal analysis of viable, early apoptotic, and late apoptotic SARS-CoV-2-specific T cells in DC-T cell cocultures as measured by Annexin-V and SYTOX AADvanced markers.

Condition	Significance	P value
**24h**		
viable tolDC-T cell vs. viable convDC-T cell	ns	>0.9999
viable tolDC-T cell vs. viable T cell alone	ns	0.9990
viable convDC-T cell vs. viable T cell alone	ns	>0.9999
early apoptosis tolDC-T cell vs. early apoptosis convDC-T cell	ns	>0.9999
early apoptosis tolDC-T cell vs. early apoptosis T cell alone	ns	0.9506
early apoptosis convDC-T cell vs. early apoptosis T cell alone	ns	0.9833
late apoptosis tolDC-T cell vs. late apoptosis convDC-T cell	ns	>0.9999
late apoptosis tolDC-T cell vs. late apoptosis T cell alone	ns	>0.9999
late apoptosis convDC-T cell vs. late apoptosis T cell alone	ns	0.9941
**72h**		
viable tolDC-T cell vs. viable convDC-T cell	ns	0.9852
viable tolDC-T cell vs. viable T cell alone	ns	0.9989
viable convDC-T cell vs. viable T cell alone	ns	0.7844
early apoptosis tolDC-T cell vs. early apoptosis convDC-T cell	ns	0.9862
early apoptosis tolDC-T cell vs. early apoptosis T cell alone	ns	0.9815
early apoptosis convDC-T cell vs. early apoptosis T cell alone	ns	>0.9999
late apoptosis tolDC-T cell vs. late apoptosis convDC-T cell	ns	0.9973
late apoptosis tolDC-T cell vs. late apoptosis T cell alone	ns	0.9667
late apoptosis convDC-T cell vs. late apoptosis T cell alone	ns	0.6619
**120h**		
viable tolDC-T cell vs. viable convDC-T cell	ns	0.9979
viable tolDC-T cell vs. viable T cell alone	ns	0.9987
viable convDC-T cell vs. viable T cell alone	ns	0.8611
early apoptosis tolDC-T cell vs. early apoptosis convDC-T cell	ns	0.8466
early apoptosis tolDC-T cell vs. early apoptosis T cell alone	ns	0.9981
early apoptosis convDC-T cell vs. early apoptosis T cell alone	ns	0.6427
late apoptosis tolDC-T cell vs. late apoptosis convDC-T cell	ns	>0.9999
late apoptosis tolDC-T cell vs. late apoptosis T cell alone	ns	0.9472
late apoptosis convDC-T cell vs. late apoptosis T cell alone	ns	0.9120

Supplementary table S4: longitudinal analysis of caspase expression by SARS-CoV-2-specific T cells in DC-T cell cocultures.

Condition	Significance	P Value
**24h**		
tolDC-T cell vs. convDC-T cell	ns	0.8468
tolDC-T cell vs. T cell alone	ns	0.9543
convDC-T cell vs. T cell alone	ns	0.6822
**72h**		
tolDC-T cell vs. convDC-T cell	ns	0.2490
tolDC-T cell vs. T cell alone	ns	0.5761
convDC-T cell vs. T cell alone	ns	0.0519
**120h**		
tolDC-T cell vs. convDC-T cell	ns	0.5092
tolDC-T cell vs. T cell alone	ns	0.7213
convDC-T cell vs. T cell alone	ns	0.1014
